# TECHNOLOGY USE IN RURAL DEMENTIA CARE: PERSPECTIVES FROM FAMILY CAREGIVERS, PWD, AND SERVICE PROFESSIONALS

**DOI:** 10.1093/geroni/igz038.3161

**Published:** 2019-11-08

**Authors:** Lucas R Prieto, Fei Sun, Wenwu Zhang, Anastasia Kononova

**Affiliations:** Michigan State University, East Lansing, Michigan, United States

## Abstract

Purpose: The use of technology in dementia care has shown promising benefits for both people living with dementia and their family caregivers, however, little is known regarding how technology is used among families affected by dementia who reside in rural communities. The purpose of this study was to explore technology use and barriers among people living with dementia, family caregivers, and service professionals who live in rural areas of Michigan. Methods: This study was based upon focus group data from six groups of family caregivers (n=32); one group of people living with early stages of dementia (n=4), and one group of service professionals (n=4) recruited from rural counties in Michigan. Results: Technology use included assisting caregiving tasks (e.g., monitoring a wandering care recipient), facilitating treatment (e.g., access treatment through telemedicine), and providing social connection and support. Themes related to strategies included addressing educational needs of young older and old-older caregivers, providing step-by-step toolkits, and collaborating with multi-sectors (e.g., public libraries, grocery stores, and churches). Discussion: Findings suggest a great need to facilitate technology literacy and competency for rural family caregivers to access caregiving resources. To effectively reach out to this population, technology methods such as local TV news network, radio, and newspaper are still beneficial. Health and social service professionals should consider collaborations with public service institutions (e.g. libraries) and faith-based organizations to include educational workshops about technology in their curriculum and training programs for dementia family caregivers.

